# The Feasibility of Robot-Assisted Chin Osteotomy on Skull Models: Comparison with Surgical Guides Technique

**DOI:** 10.3390/jcm11226807

**Published:** 2022-11-17

**Authors:** Jinyang Wu, Wenyu Hui, Jianhua Huang, Nan Luan, Yanping Lin, Yong Zhang, Shilei Zhang

**Affiliations:** 1 Department of Oral and Cranio-Maxillofacial Surgery, Shanghai Ninth People’s Hospital, Shanghai Jiao Tong University School of Medicine, College of Stomatology, Shanghai Jiao Tong University, National Center for Stomatology, National Clinical Research Center for Oral Diseases, Shanghai Key Laboratory of Stomatology, Shanghai 200011, China; 2 Department of Stomatology, Shanghai Sixth People’s Hospital, Shanghai Jiao Tong University, Shanghai 200233, China; 3 Department of Mechanical Engineering, Shanghai Jiao Tong University, Shanghai 200240, China

**Keywords:** robot, navigation, surgical guide, chin osteotomy, genioplasty

## Abstract

Surgical robotic technology is characterized by its high accuracy, good stability, and repeatability. The accuracy of mandibular osteotomy is important in tumor resection, function reconstruction, and abnormality correction. This study is designed to compare the operative accuracy between robot-assisted osteotomy and surgical guide technique in the skull model trials which simulated the genioplasty. In an experimental group, robot-assisted chin osteotomy was automatically performed in 12 models of 12 patients according to the preoperative virtual surgical planning (VSP). In a control group, with the assistance of a surgical guide, a surgeon performed the chin osteotomy in another 12 models of the same patients. All the mandibular osteotomies were successfully completed, and then the distance error and direction error of the osteotomy plane were measured and analyzed. The overall distance errors of the osteotomy plane were 1.57 ± 0.26 mm in the experimental group and 1.55 ± 0.23 mm in the control group, and the direction errors were 7.99 ± 1.10° in the experimental group and 8.61 ± 1.05° in the control group. The Bland–Altman analysis results revealed that the distance error of 91.7% (11/12) and the direction error of 100% (12/12) of the osteotomy plane were within the 95% limits of agreement, suggesting the consistency of differences in the osteotomy planes between the two groups. Robot-assisted chin osteotomy is a feasible auxiliary technology and achieves the accuracy level of surgical guide-assisted manual operation.

## 1. Introduction

Precise and stable osteotomy, reduction, and fixation are of great importance for reconstructing maxillofacial function and appearance and protecting adjacent vital anatomical structures during craniomaxillofacial bone surgery. As digital surgical techniques have rapidly advanced recently, 3D virtual surgical planning (VSP) [1,2,3], computer-aided design and manufacturing (CAD/CAM) [4], image-guided surgical navigation [5], virtual reality and augmented reality [6,7], and surgical robotics [8,9] have been reported to have high precision.

Among these techniques, the surgical guides based on CAD/CAM combined with rapid prototyping or 3D printing techniques can assist in osteotomy, drilling, bone-segment positioning, and bone-segment modeling, in which preoperative VSP can be transferred into the real surgical process. In recent years, surgical robotic technology, characterized by its high accuracy, good stability and repeatability, the ability to decrease work intensity, avoid tremor and fatigue that can occur with human hands, and better meet the needs of minimally invasive surgery, has become a focus of research and application in digital surgical techniques. However, this technique has been rarely reported in the field of oral and cranio-maxillofacial bone surgery.

The present study intends to examine the feasibility of the self-developed craniomaxillofacial surgery robotic system (CMF ROBOT) in chin osteotomy, analyze its operative accuracy via comparison with a surgical guide, and preliminarily assess its osteotomy efficacy.

## 2. Materials and Methods

### 2.1. Establishment of the CMF ROBOT System

**Figure 1:**
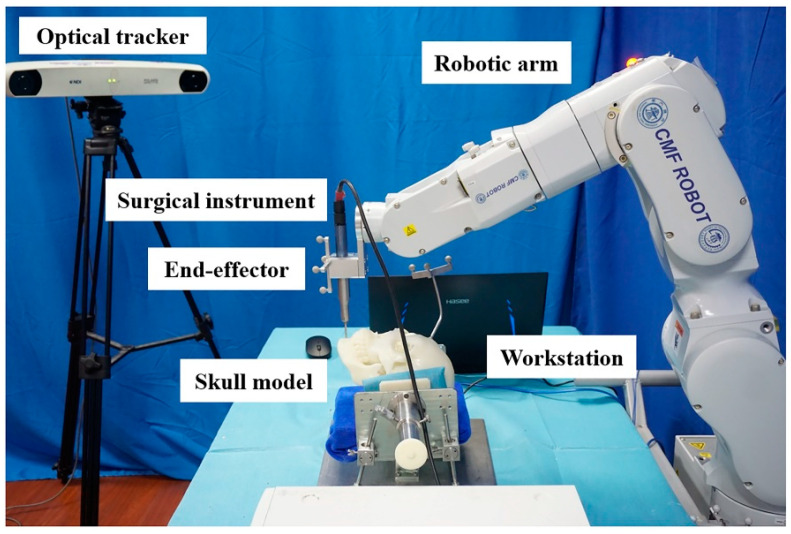
Structure of the CMF ROBOT system.

The cranio-maxillofacial surgical robotic system, termed the CMF ROBOT system, was independently developed by our group based on clinical use and testing needs (Figure 1) [10]. The hardware system consists of a six-degree-of-freedom C8L robotic arm (EPSON Robots, “Long Beach, CA, USA), an individually designed end-effector, and a Polaris Spectra optical tracker (Northern Digital Inc., Waterloo, ON, Canada). The software system consists of the self-developed robotic software CMF Robot Plan 1.0, which can perform functions such as preoperative 3D VSP, surgical trajectory planning, and intraoperative navigation. The parameters of the robotic system are as follows: working radius: 901 mm, accuracy of repeated positioning: 0.05 mm, maximum load: 8 kg, spatial optical tracking accuracy: 0.25 mm, and sampling frequency: 60 Hz. Stryker System 5 (Stryker Corporation, Kalamazoo, MI, USA) was used as the surgical power system for the end-effector, and its micro-reciprocating saw met the needs for the osteotomy in this model experiment.

### 2.2. Preoperative Design and Skull Model Fabrication

In this study, the spiral computed tomography (CT) images of 12 patients with dentofacial deformities (four cases of skeletal class I malocclusion, four cases of skeletal class II malocclusion, and four cases of skeletal class III malocclusion) were imported into the design software, Geomagic Studio 12.0 (Geomagic Inc., Morrisville, NC, USA), in the form of DICOM data to construct a 3D skull model. The landmark points for navigation registration were set on the model in the virtual environment of the software. The 12 skull models were subjected to chin osteotomy during preoperative VSP, and the osteotomy plane was determined by classical horizontal osteotomy. First, the position and orientation of the inferior alveolar nerve canals in the mandible were traced in each skull model, and the 3D image of the complete dentition was reconstructed according to the dental image threshold. Then, the intersection line between the osteotomy plane and the surface of the mandible on the labial side was designed on the surface of the mandible at 5 mm below the mental foramens on both sides, with the osteotomy plane parallel to the occlusal plane. The 3D virtual skull models designed were saved in the stereolithography (STL) format and then imported by an Objet260 Connex3 3D printer (Stratasys Ltd., Eden Prairie, MN, USA). The data were used to construct a total of 24 real skull models using MED620 resin materials for 3D printing (Stratasys Ltd., MN, USA); two prints were performed for each model. This study was approved by the institutional review board and the ethics committees of Shanghai Ninth People’s Hospital, Shanghai Jiao Tong University School of Medicine (2016-161-T110).

### 2.3. Procedures of Chin Osteotomy

#### 2.3.1. Experimental Group: Robot-Assisted Chin Osteotomy

**Figure 2:**
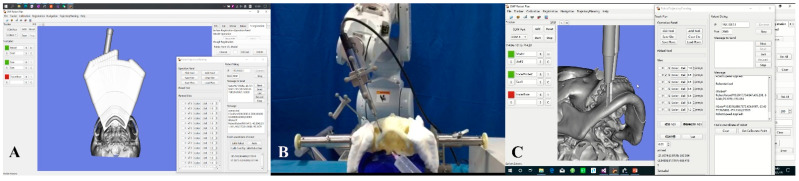
Robot-assisted chin osteotomy. (**A**) The robot’s surgical trajectory was planned based on the osteotomy plane determined by VSP. The virtual reciprocating saws were set parallelly along the osteotomy plane. (**B**) The robot was automatically performing the chin osteotomy. (**C**) Synchronous screenshot of the software showed the robot-assisted chin osteotomy.

Preoperative preparation: The designed 3D virtual skull model was imported with the robotic software, CMF Robot Plan 1.0, in the STL format as the target of the chin osteotomy. The robot’s surgical trajectory was planned based on the osteotomy plane determined by VSP (Figure 2A). A set of forward planning points was set on the osteotomy line at an interval of 2 mm, while a set of pulling planning points (sinking and lifting) was also set to simulate the hand movements of surgeons during osteotomy. This technique had the following advantages: (1) Additional planning points increased the opportunity for the robot to correct the real-time position during the operation, and the operative accuracy of the robot could be guaranteed through ideal preoperative trajectory planning in the software. (2) The pulling action of surgeons was simulated during surgical trajectory planning so that the reciprocating saw installed on the end-effector could have an enhanced cutting efficiency.

Surgical process: The dynamic reference frame for optical navigation was mounted on the right frontal bone of the skull model, and the landmark points on the model were selected through point registration to complete the system registration. With the navigation tracker mounted on the fixture of the end effector, the navigation system could calibrate the reciprocating saw on the fixture of the end effector through the tip of the navigation probe. Then, the robot automatically moved to the preoperatively designed position along the osteotomy plane after receiving the surgical planning orders from CMF Robot Plan 1.0, thereby completing the chin osteotomy (Figure 2B,C).

The preparation time, including the preoperative trajectory planning and navigation system preparation, and the operating time from the beginning of the robot-assist osteotomy to the end, were recorded.

#### 2.3.2. Control Group: Surgical Guide-Assisted Osteotomy

**Figure 3:**
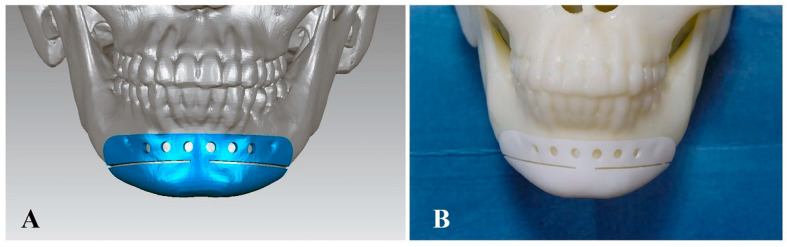
Surgical guide-assisted osteotomy. (**A**) Design of the surgical guide. (**B**) The 3D-printed surgical guide was placed on the skull model.

Preoperative preparation: The osteotomy guide was designed on the 3D virtual skull model using the design software Geomagic Studio 12.0 according to the chin osteotomy plane generated by VSP. The edge of the bone-supported guide was designed to wrap the mandibular edge so that the only positioning information of the guide could be provided by the bone surface (Figure 3A). Finally, the virtually designed surgical guide was imported into a 3D printer (ProJet 3510, 3D system, Rock Hill, SC, USA) in the STL format and printed with resin materials to form a real surgical guide.

Surgical process: After the 3D-printed surgical guide was stably placed on the skull model (Figure 3B), an experienced surgeon completed the chin osteotomy with a reciprocating saw according to the positioning information of the guide.

The preparation time, including preoperative guide design and intraoperative guide fixation, and the operating time from the beginning of the manual osteotomy to the ending, were recorded

### 2.4. Assessment Methods

**Figure 4:**
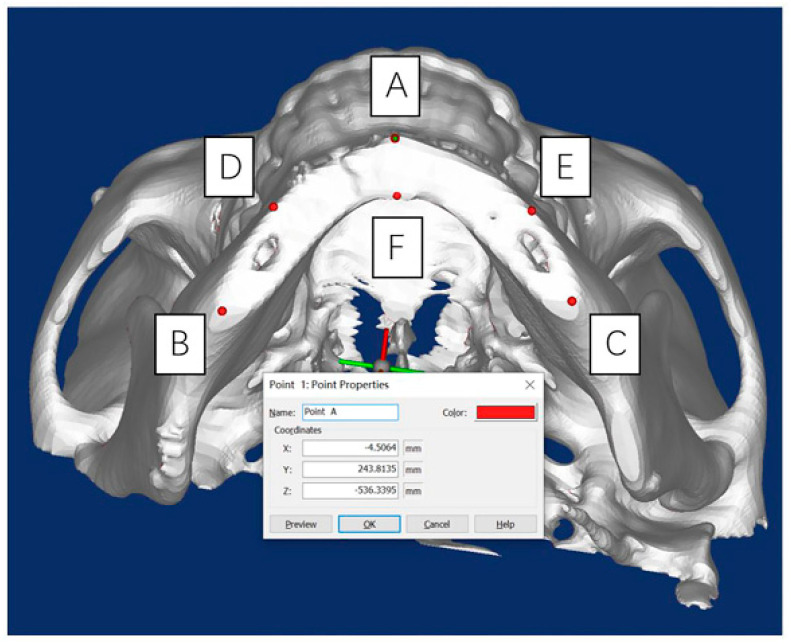
The locations of measuring points A, B, C, D, E, and F.

Spiral CT scans were conducted on the skull model in the experimental group and control group after the operation, followed by 3D reconstruction using Geomagic Studio 12.0. The STL files of the 3D virtual skull model after the operation and those determined by preoperative VSP were subjected to spatial registration because one plane can be determined by three points in space. Point A (x_a_, y_a_, z_a_) is located at the edge of the buccal bone surface on the osteotomy plane and intersects with the median sagittal plane. The intersection points B (x_b_, y_b_, z_b_) and C (x_c_, y_c_, z_c_), between the osteotomy plane and the bilateral mandibular edges, were used to determine the corresponding osteotomy plane (Figure 4).

At this time, using point A as an example, the spatial distance between the preoperative simulation and postoperative outcome at the same point could be calculated.

**Figure 5:**
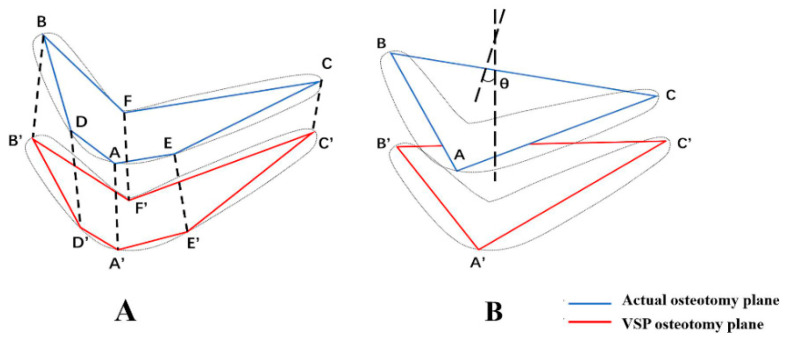
Diagram illustrating the measurement of distance error and the direction error of the osteotomy plane. (**A**) Distance error. (**B**) Direction error.

Points with clear anatomical positions on the irregular actual osteotomy plane and the virtual planned osteotomy plane could be connected to approximate two polygons, and the spatial distance of the two planes was expressed as the mean value of the Euclidean distances of the vertices of the two planes. In addition to points A, B, and C, points D (x_d_, y_d_, z_d_) and E (x_e_, y_e_, z_e_), located at the edge of the buccal bone surface on both sides of the mandible and intersecting with the vertical line of the mental foramen, and point F (x_f_, y_f_, z_f_), located at the edge of the lingual bone surface on the osteotomy plane and intersecting with the median sagittal plane, were obtained. Then, the mean value of the Euclidean distances between the six points and the corresponding points in the 3D virtual design image were calculated and used to describe the distance error between the postoperative osteotomy outcome surface and the VSP (Figure 5A).

In addition, the normal vector in the 3D space of the osteotomy plane was calculated using MATLAB R2020b (MathWorks Inc., Natick, MA, USA).

Similarly, the normal vector in the space of the osteotomy plane of the skull model determined by preoperative VSP was calculated. Then, the angle difference θ between the normal vectors of the two planes was obtained to describe the direction error between the postoperative osteotomy plane and the VSP plane (Figure 5B).

### 2.5. Statistical Analysis

SPSS 22.0 (IBM, Armonk, NY, USA) was used for the statistical analysis of the distance error and direction error of the osteotomy plane between the experimental group and the control group. The consistency between the two groups was determined through paired *t*-tests, the intraclass correlation coefficient (ICC), and Bland–Altman analysis. *p* < 0.05 was considered statistically significant.

## 3. Results

**Figure 6:**
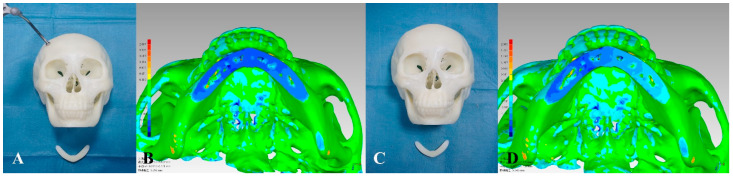
Surgical outcomes. Experimental group: (**A**) Actual osteotomy. (**B**) Image fusion showed the comparison of mandibular osteotomies between the postoperative CT and VSP. Control group: (**C**) Actual osteotomy. (**D**) Image fusion showed the comparison of mandibular osteotomies between the postoperative CT and VSP.

The surgical procedures were successfully completed 24 times on the skull models (Figure 6).

In the experimental group, the surgical robotic system worked normally, and no mechanical failure or malfunction occurred. The overall distance error of the osteotomy plane was (1.57 ± 0.26) mm, and the direction error was (7.99 ± 1.10)° (Table 1). The preoperative preparation lasted 38.0 min (33–43 min) on average. The robot spent 8.5 min (7.7–9.4 min) on average on the osteotomies. In the control group, the surgical guide was accurately positioned and worked smoothly. The overall distance error of the osteotomy surface was (1.55 ± 0.23) mm, and the direction error was (8.61 ± 1.05)° (Table 1). The preoperative preparation lasted an average of 27.8 min (22–35 min). The average duration of the manual osteotomy was 4.1 min (2.8–6.4 min).

**Figure 7:**
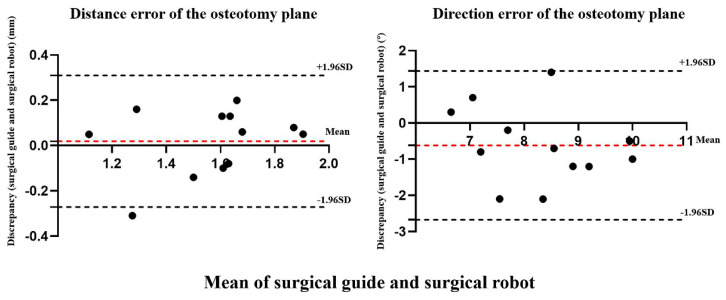
The Bland–Altman plot of distance error and direction error between the surgical guide and surgical robot.

According to the statistical results, the distance error of the osteotomy plane was not significantly different between the experimental and control groups (*p* = 0.66 > 0.05) and showed good reliability (ICC = 0.819 > 0.75). The direction error of the osteotomy plane was not significantly different between the two groups (*p* = 0.07 > 0.05) and showed moderate reliability (0.4 < ICC = 0.630 < 0.75) (Table 2). The Bland–Altman analysis results revealed that the distance error of 91.7% (11/12) and the direction error of 100% (12/12) of the osteotomy plane were within the 95% limits of agreement, suggesting the consistency of the differences in the osteotomy planes between the two groups (Figure 7).

## 4. Discussion

Accurate bone surgery is essential for functional reconstruction and morphological recovery in the treatment of oral and craniomaxillofacial abnormalities or osseous diseases. The surgical guide technique allows for the precise and stable application of preoperative VSP in actual surgical procedures and therefore has been widely used in clinical practice [11]. However, it also has some shortcomings. First, for currently marketed surgical guides, deformation may arise during the processing and disinfection of resin materials, affecting accuracy. Second, in the event of intraoperative rupture, the surgical guide will no longer be available. Third, to ensure the retention of the surgical guide, it is necessary to separate and expose a large area of bone surface relative to the target surgical region, causing additional surgical injury. Therefore, there is a necessity to identify a more ideal high-precision osteotomy technique.

Based on the premise of guaranteed accuracy, surgical robotic technology is more stable during surgery, is minimally invasive, and avoids human hand tremor and fatigue. This technology has recently been applied to multiple surgical fields (e.g., orthopedics, ophthalmology, neurosurgery, urology, and thoracic surgery). In the field of oral and cranio-maxillofacial surgery, robotic technology is mostly utilized in soft tissue surgery associated with neoplasms [8,9,12,13] and dental implant surgery [14,15]. Regarding theoretical research and experimental testing, the application of this technology is centered on orthognathic surgery [10,16,17,18,19], mandibular angle split osteotomy [20,21,22], and fibula preparation for mandibular reconstruction [23,24,25]. Nonetheless, the application of robotic surgical procedures in oral and cranio-maxillofacial bone surgery is restricted to drilling holes [14,26,27,28,29], guiding manual osteotomies after drilling several guide paths [19,20,21,22], or the simple positioning of bone segments [17,18,24]. Reports of robotic technology directly applied in oral and craniomaxillofacial osteotomy are rare [10,16,25]. Hence, this study aimed to investigate the feasibility of robot-assisted chin osteotomy.

Currently, the operative accuracy of surgical robot-assisted osteotomy in bone surgery is mainly evaluated from the following two aspects. First, the operative outcome of the robot and the preoperative VSP are compared to determine the operative accuracy. In a study, robot-assisted genioplasty was performed for six patients, which showed a distance error of (2.19 ± 0.48) mm from the actual osteotomy line, indicated by postoperative CT, to the osteotomy surface determined by preoperative VSP [19]. In another study, the authors conducted 18 osteotomy experiments on three fibula models using the KUKA surgical robot, and the image fusion of postoperative CT and VSP images confirmed that the average linear distance error and the average angular error of the osteotomized segments were (1.3 ± 0.4) mm and (4.2 ± 1.7)°, respectively [23]. Second, the operative outcomes are compared between the surgical robot and other types of digital technology-assisted manual operation. An orthognathic surgery-related robot-assisted osteotomy was carried out on a skull model and found that the results were not significantly different from those of navigation-guided osteotomy regarding osteotomy error, indicating the consistency of the techniques [10]. In an animal experiment, surgeons performed mandibular angle osteotomy under the real-time guidance of a surgical robot, and based on the comparison with preoperative VSP, the osteotomy error was significantly less in the robot group than that in the manual operation group [30].

The two ideas above inspired the comparison of surgical robotic technology and the commonly used surgical guide technique for chin osteotomy in the present study. The osteotomy effects of both groups were compared with those of preoperative VSP, and the operative deviation in 3D space was evaluated using the distance error and direction error of the osteotomy plane. The experimental results indicated that the robotic system successfully completed the osteotomy, and the distance error of the osteotomy plane was (1.57 ± 0.26) mm. The authors clinically investigated 62 patients undergoing orthopedic robot-assisted unicompartmental knee arthroplasty and proposed that the precision of the osteotomy plane for a surgical robot should reach 1.0–1.5 mm to ensure the surgical effect [31]. Nonetheless, in most existing reports on the accuracy of osteotomy by a surgical robot, precision within 2 mm is considered to meet clinical requirements [10,20,22,32,33,34]. Moreover, the direction error of the osteotomy plane in robot-assisted chin osteotomy should be (7.99 ± 1.10)°. Although no studies have reported its assessment criterion, this indicator provides a scientific assessment of the spatial error of the osteotomy plane. Under the same conditions, compared with manual operation by experienced surgeons with the aid of surgical guides, robot-assisted chin osteotomy showed no statistically significant difference in these two indicators, and Bland–Altman analysis validated that the error of the osteotomy plane was consistent between the two groups. Based on previous research and the current experimental findings, the CMF ROBOT system can be considered to have favorable operative precision and an accuracy level that is consistent with that of surgical guide-assisted manual operation.

Although the surgical robot group had a longer preoperative preparation time and operation time than the surgical guide group, the robotic system can automatically complete the routine osteotomy, which can prevent energy and physical expenditure by surgeons during the operation and decrease the intraoperative risks associated with the unstable control of surgical instruments due to hand fatigue. Robot-assisted surgery is an evolution of traditional surgical procedures. As artificial intelligence is applied and researched for preoperative VSP and robotic trajectory planning, the preparation time for robot-assisted surgery is expected to decrease.

However, some limitations were also identified in this study. First, certain systematic errors were inevitably accumulated by the current experimental method during the spiral CT scans, 3D image reconstruction, and spatial registration of the skull models, which were included in the operative accuracy deviation of the osteotomy. However, the same measurement method was used in the experimental and control groups; therefore, the consistency between the two groups was not affected by these systematic errors. In subsequent studies, methods to decrease the systematic error by optimizing measurement methods will need to be addressed. Furthermore, the skull model experiment did not involve the soft tissues around the bones, and the mechanical properties of the resin materials differed from those of real bone tissue. Therefore, it is necessary to test the properties of the CMF ROBOT system through further animal and cadaver experiments, thus laying theoretical and experimental foundations for clinical application. Juergens and his research group completed a series of experimental and clinical studies with robot-guided laser maxillo-facial osteotomies, and this technique was applied in midface osteotomies during orthognathic surgery in the clinics [35,36]. Robot-assisted osteotomy is not far away from us.

## 5. Conclusions

Robot-assisted chin osteotomy is a feasible auxiliary technology in oral and cranio-maxillofacial surgery. The CMF ROBOT system can complete chin osteotomies as per the preoperative VSP. Effective measurement and evaluation methods demonstrated that this technology has good stability and accuracy and achieves the accuracy level of surgical guide-assisted manual operation. Robotic surgery can be accurate on a genioplasty on an ex vivo skull model. In addition, the surgical robotic system can automatically complete the chin osteotomy. Surgical robotic technology can decrease the energy and physical expenditure of the surgeon during the operation and improves the traditional surgical operation mode. In the future, robot-assisted osteotomies may be applied to the cranium, zygoma, maxilla, and other sites for procedures such as cranial osteotomy, malar plasty, and maxillary osteotomy, but its efficacy and safety need to be validated by additional experimental tests and clinical procedures.

## Figures and Tables

**Table 1:** The operative results.

	Experimental Group		Control Group	
No	Distance Error (mm)	Direction Error (°)	Distance Error (mm)	Direction Error (°)
1	1.14	9.7	1.09	8.3
2	1.37	8.6	1.21	8.6
3	1.71	9.5	1.65	7.9
4	1.56	8.3	1.66	9.0
5	1.59	7.3	1.67	8.3
6	1.91	6.8	1.83	8.8
7	1.93	6.5	1.88	9.2
8	1.67	6.8	1.54	8.7
9	1.43	9.2	1.57	8.2
10	1.70	7.6	1.57	9.1
11	1.12	7.4	1.43	9.2
12	1.76	8.2	1.56	8.1
Mean ± SD ^1^	1.57 ± 0.26	7.99 ± 1.10	1.55 ± 0.23	8.61 ± 1.05

^1^ SD, Standard Deviation.

**Table 2:** Paired *t*-tests of operative errors between the robot-assisted osteotomy and surgical guide-assisted osteotomy.

Paired Groups	Measurement	Mean	SD ^1^	SEM ^2^	95% CI ^3^		*p* Value
					Lower Limit	Superior Limit	
Experimental group	Distance error (mm)	0.02	0.15	0.04	−0.08	0.11	0.66
Control group	Direction error (°)	−0.62	1.05	−0.30	−1.28	0.05	0.07

^1^ SD, Standard Deviation. ^2^ SEM, standard error of mean. ^3^ CI, confidence interval. *p* > 0.05 was considered no statistical significance between the experimental group and the control group.

## Data Availability

The data presented in this article are available within this article.
